# Intrahepatic Biliary Cystadenoma With Colonic Adenomatous Polyps in a Patient With Chronic Hepatitis B: A Case Report and Literature Review

**DOI:** 10.3389/fmed.2021.760607

**Published:** 2021-12-16

**Authors:** Yan Tang, Chenyu Wang, Shunjun Fu, Ting Li, Guolin He

**Affiliations:** ^1^The Remarkable and Innovation Class, The Second School of Clinical Medicine, Southern Medical University, Guangzhou, China; ^2^Department of Hepatobiliary Surgery II, Zhujiang Hospital, Southern Medical University, Guangzhou, China

**Keywords:** liver, biliary cystadenoma, cystic liver disease, colon adenomatous polyps, chronic hepatitis B, intraductal papillary neoplasms of the bile duct

## Abstract

**Background:** Biliary cystadenomas are rare cystic tumors of the bile duct system that are mostly benign but also have the possibility of malignant transformation. Biliary cystadenomas mostly occur in the intrahepatic bile ducts and are more common in middle-aged women. Due to non-specific radiology, preoperative diagnosis is difficult and is usually performed by postoperative pathology. Complete resection is the best treatment option, and the postoperative prognosis is good.

**Case Description:** This study reports a case of a patient with biliary cystadenoma who was diagnosed with simultaneous chronic hepatitis B and colon (hepatic flexure) adenomatous polyps. The patient presented to the doctor because of abdominal pain, and a blood test showed hepatitis B. Computed tomography revealed both right liver and colonic lesions. Colonoscopy revealed polyps, and the postoperative pathological diagnosis was adenomatous polyps. Laparoscopic resection of the right liver tumor was performed, and it was diagnosed as hepatobiliary cystadenoma by postoperative pathological analysis combined with immunohistochemistry.

**Conclusion:** In patients with chronic hepatitis, the shape of biliary cystadenoma may not be very typical, and it is necessary to combine this with immunohistochemistry for diagnosis. When multiple lesions are detected in the painful area, the diagnosis of each lesion and its treatment sequence are worthy of consideration. Under normal circumstances, the prognosis of biliary cystadenoma is good; however, in patients with chronic hepatitis B, more cases need to be observed for verification.

## Introduction

Biliary cystadenomas are rare cystic tumors of the biliary system, most of which are benign but may also be malignant (1). Biliary cystadenomas mostly occur in the intrahepatic bile duct (90%) and are rare in the extrahepatic bile duct system and gallbladder (2, 3). Biliary cystadenomas mainly occur in women (>85%), with an average age of 45 years (1, 4). Most patients with biliary cystadenoma are asymptomatic, and some may show a wide range of symptoms, the most common of which are abdominal pain and distension (5). Because the radiological characteristics are non-specific, the preoperative diagnosis is challenging, and the diagnosis is confirmed by pathology after the lesion is removed (6). Due to the potentially malignant degeneration of these lesions, treatment must be performed as extensively as possible through surgery (7).

To date, it has not been reported that patients with chronic hepatitis B were diagnosed with biliary cystadenoma at the same time and, more particularly, combined with colonic (hepatic flexure) adenomatous polyps. This study reports on such a case.

## Case Presentation

Due to dull pain in the right upper abdomen for ~1 month, a 46-year-old woman was admitted to a local hospital, in which computed tomography (CT) revealed space-occupying lesions of the liver. She was then admitted to our hospital for further treatment. The patient reported that pain in the right upper abdomen did not radiate to other areas, with no headache, dizziness, chest tightness, shortness of breath, nausea and vomiting, diarrhea, black stool, frequent urination, urgency, and other discomforts. She had no history of any major diseases, and her family and social histories were non-significant.

Her physical examination showed no abnormalities. The admission blood test showed that the indirect bilirubin level was 3.2 (normal, 5.1–13.7) μmol/L, activated partial thromboplastin time was 23.6 (normal, 25.0–31.3) s, and carcinoembryonic antigen and carbohydrate antigen levels were within the normal range. The following were also noted: quantitative hepatitis B virus DNA (HBV DNA) level, 1.12 × 107 (positive) IU/mL; quantitative hepatitis B surface antigen (HBsAg-C) level, 2,675.000 cut-off index (COI) (normal, <1.00); quantitative hepatitis B e antigen (HBeAg-C) level, 1,430 COI (normal, <1.00); and quantitative hepatitis B core antibody (HBcAb-C) level, 0.007 COI (normal, >1.00), which were suggestive of chronic hepatitis B.

**Figure 1:**
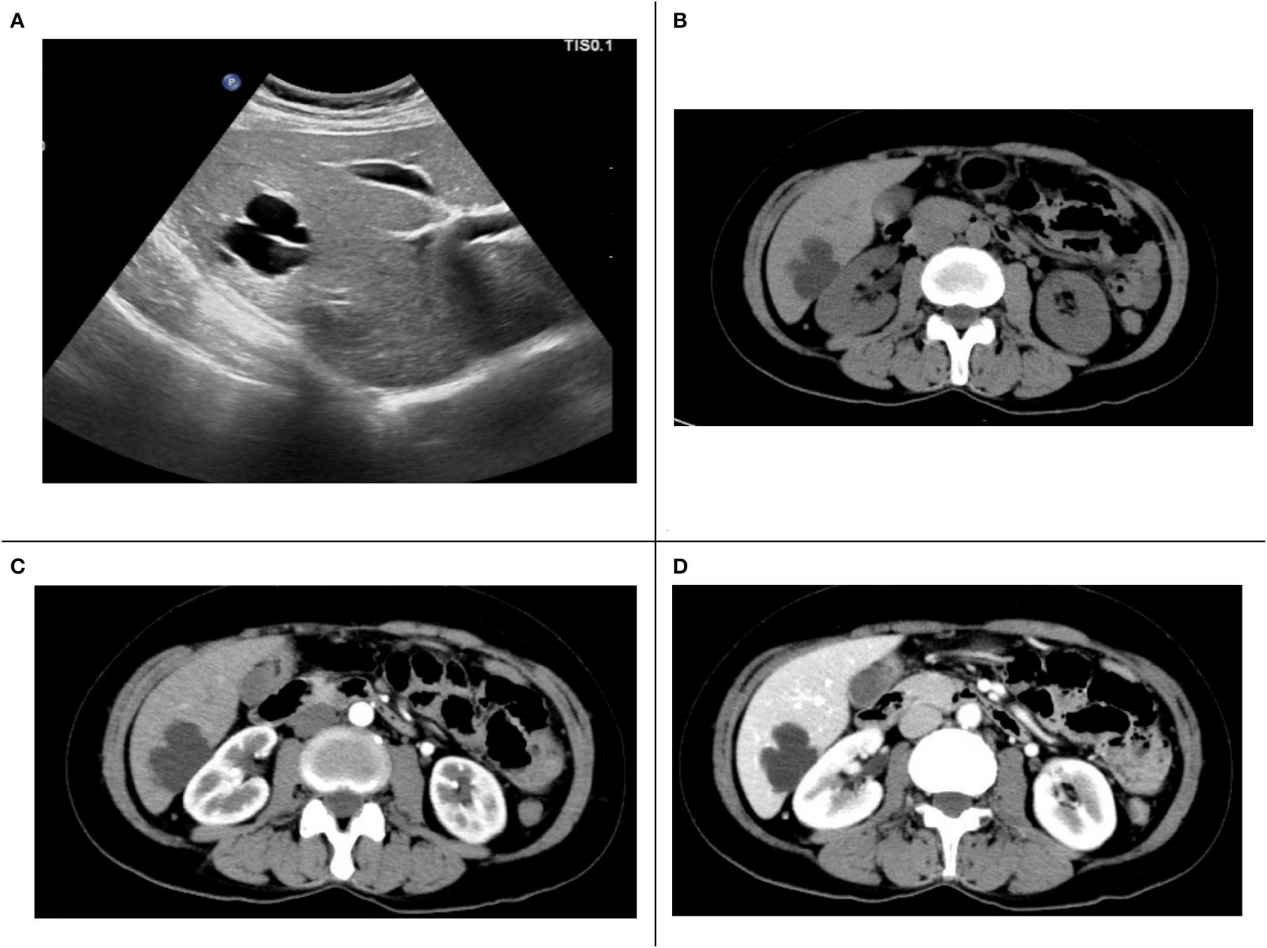
**(A)** Liver ultrasound. **(B)** Computed tomography (CT) plain scan. **(C)** The arterial phase of enhanced CT scans. **(D)** The portal phase of enhanced CT scans.

Ultrasound examination of the abdomen showed cystic liver lesions, ~38 × 28 and 13 × 8 mm in sizes, and separated light bands could be seen in the larger ones (Figure 1A). CT of the abdomen revealed multiple cysts in the liver; the larger ones were in the shape of a rosette and located in the lower part of the right lobe of the liver; the largest cross-sectional area was ~37 × 31 mm, and there seemed to be a separate structure inside (Figure 1B). In the arterial phase, the subcapsular enhancement focus of the lower right anterior lobe of the liver was enhanced (Figure 1C), which is unclear in the portal phase (Figure 1D) and cavernous hemangioma was considered. CT also showed that the intestinal wall of the liver flexure of the colon was thickened and significantly strengthened, which must be diagnosed by colonoscopy.

**Figure 2:**
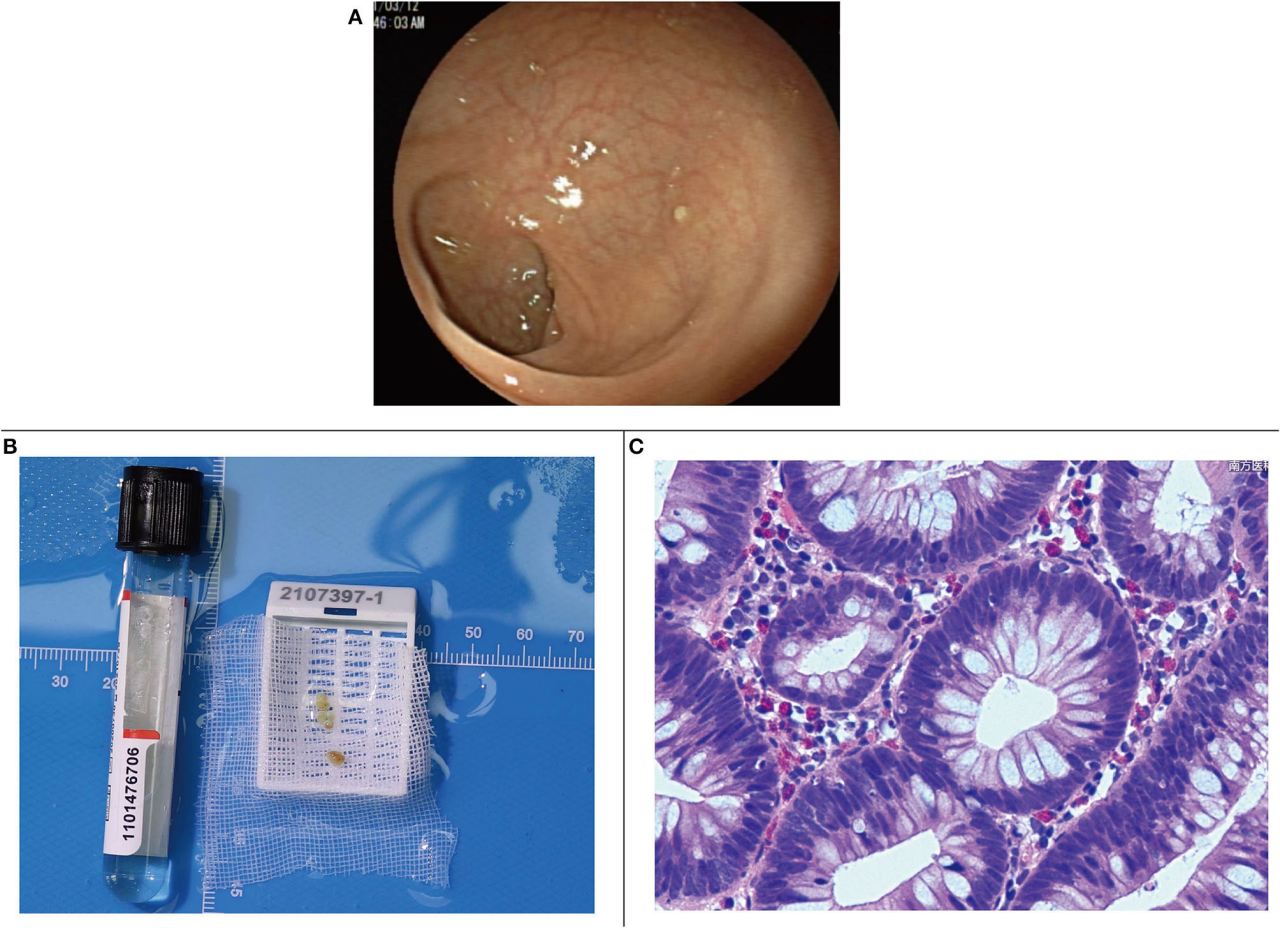
**(A)** Colonic liver flexure polyps seen by endoscopy. **(B)** Gross specimen of colonic liver flexure polyps. **(C)** Microscopic image of colonic liver flexure polyps.

**Figure 3:**
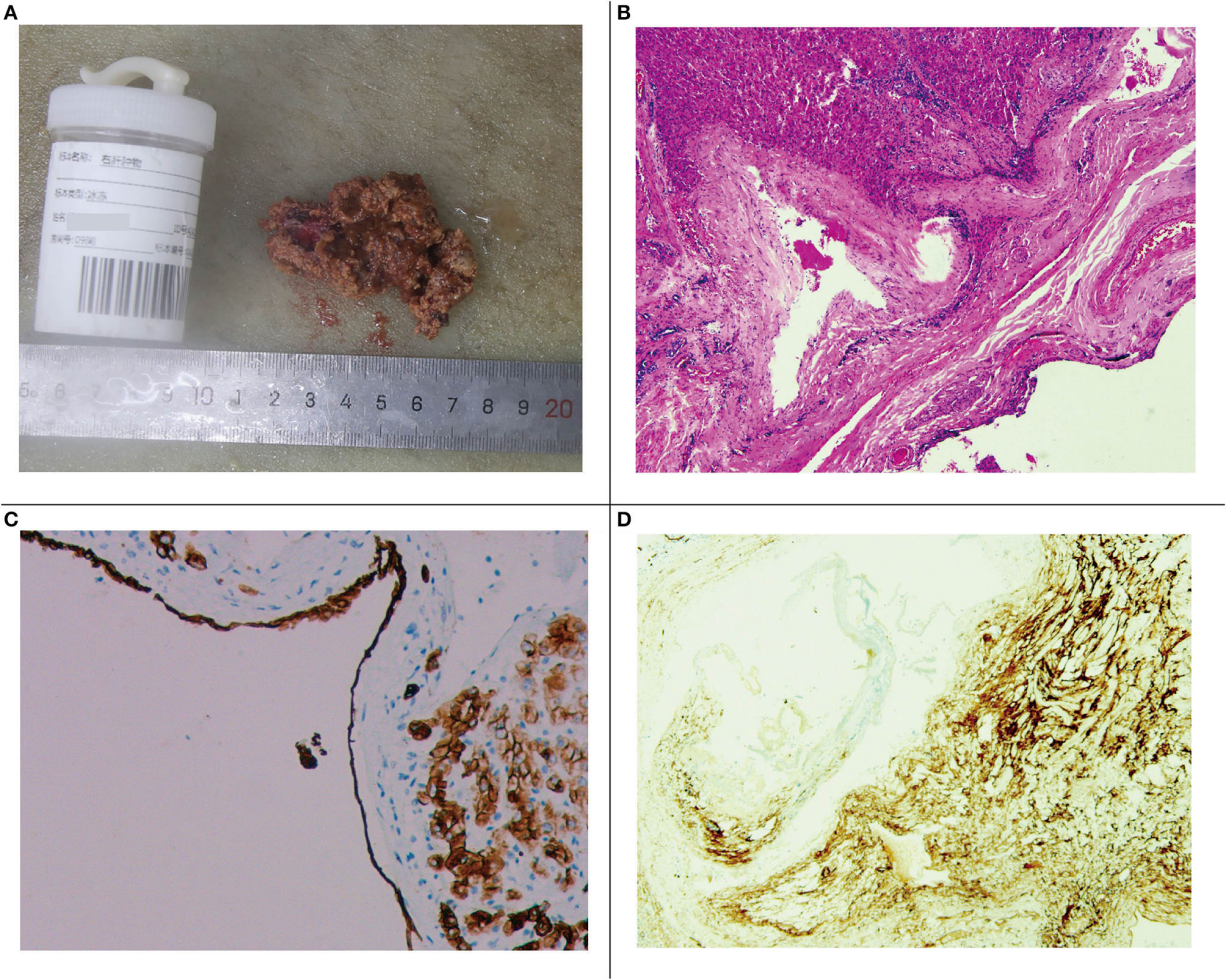
**(A)** Gross specimen of the right liver tumor. **(B)** Microscopic image of the right liver tumor. **(C)** Immunohistochemical staining of the cyst gland duct epithelium (CK+). **(D)** Immunohistochemical vascular staining (CD34+).

Under electronic colonoscopy, three flat polyps sized 2–4 mm were found in the liver flexure of the colon (Figure 2A), all of which were clamped. Macroscopically, the pathological tissue was four pieces of gray-white tissue with a size of 0.5 × 0.4 × 0.3 cm (Figure 2B), of which the pathological diagnosis was adenomatous polyps (Figure 2C). Laparoscopic resection of the right liver tumor was performed the following day. During the surgery, we observed a vascular mass-like nodule protruding from the surface of the right liver near the right posterior side of the gallbladder, soft in quality, and obvious adhesion to the surrounding area. Ultrasound guidance was used repeatedly during the surgery to completely remove the liver tumor. Gross examination of the surgical specimen showed a gray-red liver tissue with an overall size of 5.5 × 4 × 0.8 cm. The cut surface was gray-red, and the surgical specimen was solid and soft, with no obvious nodules (Figure 3A). The microscope showed that the structure of the liver lobules was basically normal, the liver cells were focally degenerated, focal hyperplastic ducts were observed, and most of the cystic structures were not clearly lined with the epithelium (Figure 3B). Combined with the results of immunohistochemistry (CK+, CD34+), it is consistent with biliary cystadenoma (Figures 3C,D).

**Figure 4:**
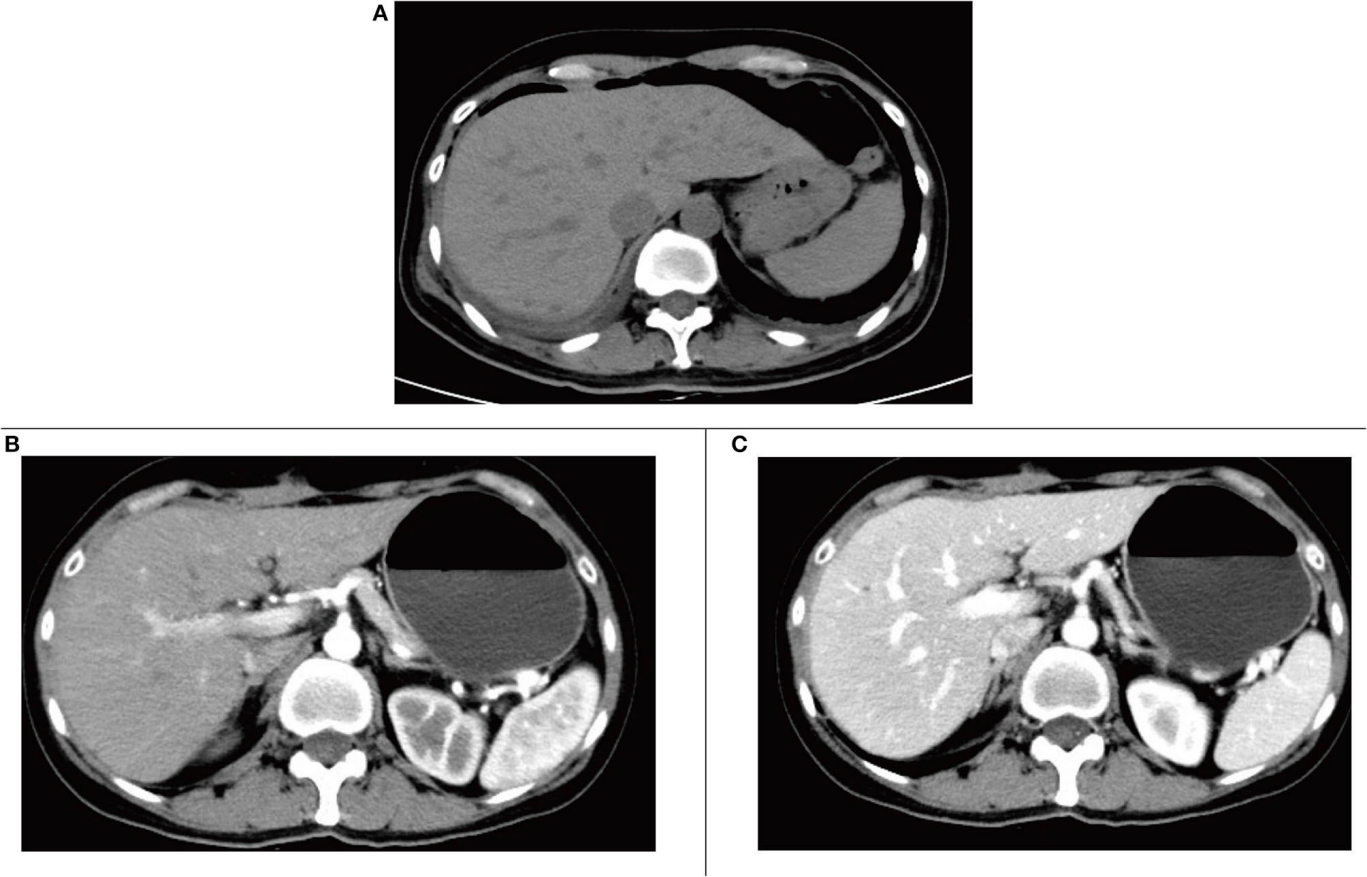
**(A)** Computed tomography (CT) plain scan after surgery. **(B)** The arterial phase of enhanced CT scan 2 months after surgery. **(C)** The portal phase of enhanced CT scan at postoperative 2 months.

Postoperative CT showed resection of the posterior lower segment of the right liver lobe; a small amount of fluid, blood, and gas in the residual cavity; drainage tube indwelling; and a small amount of fluid around the gallbladder fossa (Figure 4A). Re-examination at postoperative 2 months revealed changes in CT findings after resection of the posterior lower segment of the right liver lobe and cystic fluid in the residual cavity, and a small amount of fluid around the original gallbladder fossa was absorbed and dissipated (Figures 4B,C).

## Discussion

Most patients with biliary cystadenoma are asymptomatic, and such patients are usually diagnosed incidentally during imaging examinations or surgical exploration (8). Patients who visit a doctor with symptoms usually complain of abdominal discomfort, the most common being abdominal pain and distension (5). Patients with biliary cystadenoma may also develop obstructive jaundice, cholangitis, hemorrhage, and cyst rupture under certain rare circumstances (8). The patient in this case visited the hospital because of dull pain in the right upper abdomen for 1 month, and to a large extent, it can be explained by the biliary cystadenoma lesion of the right liver. Adenomatous polyps were found in the liver flexure of the colon, and the contribution of these lesions to the symptoms of dull pain in the upper right abdomen is worth discussing. Most polyps do not cause any pain until they are large enough to cause colonic obstruction; therefore, colonic polyps in this patient were not large enough to cause pain by obstructing the colon. As for chronic hepatitis B, since the patients with this disease are generally asymptomatic and the liver function of the patient in this case was normal, it is considered that it did not contribute to the symptoms of abdominal pain in this case.

However, our examination was not comprehensive regarding the cause of the patient's upper abdominal pain. Upper gastrointestinal endoscopy should be performed to rule out other causes of right upper abdominal pain.

Biliary cystadenoma is usually a multilocular, isolated lesion with liquid content (8). Under light microscopy, biliary cystadenoma consists of three layers: cyst lining of the bile duct epithelium, medium to dense intercellular substance, and dense collagen connective tissue layer (5). However, the pathological report of this patient showed focal hyperplastic ducts, and most of the cystic structures were not clearly lined with the epithelium (Figure 3B). Compared with the results in other case reports, the results under light microscopy in this case are not typical, and only the results of immunohistochemistry suggest biliary cystadenoma (Figures 3C,D). We believe that there are two possible influencing factors. One is that chronic hepatitis B leads to the atypical development of biliary cystadenoma, and the other is that the surgical method of laparoscopic liver tumor resection may cause some damage to the morphology of the specimen.

The diagnosis and differential diagnosis of cystadenoma mainly rely on abdominal ultrasound, CT, and magnetic resonance imaging (MRI). CT and enhancement have advantages in displaying the anatomy and blood vessels of the lesion, but ultrasound shows that the internal separation of the cyst is more obvious, which also proves that ultrasound and CT are complementary (9). Compared with CT, MRI can show the anatomical relationships in the liver to a higher degree, which helps to plan surgery (10). MRI was not performed in this case because it was relatively expensive and time-consuming, and it seemed that it was not of high value to further confirm the diagnosis at that time. We used multiple ultrasound guidance during the surgery, which can achieve the goal of complete resection of the lesion.

Differential diagnoses include simple liver cysts, parasitic cysts (especially hydatid cysts), hematomas or post-traumatic cysts, liver abscesses, congenital cysts, and other cystic liver diseases, while biliary cystadenoma and cystadenocarcinoma can only be accurately distinguished by postoperative histopathological evaluation (11). The condition of this patient was misdiagnosed as a cavernous hemangioma, mainly because the arterial phase showed subcapsular enhancement. In addition, we did not fully consider the differential diagnosis of cavernous hemangioma and biliary cystadenoma before surgery, which is one of the limitations of our diagnosis and treatment process.

Complete resection is the first treatment for biliary cystadenoma. Other treatment options such as internal drainage, aspiration, marsupialization, sclerosis, Roux-en-Y cyst-bowel anastomosis, or partial resection are associated with complications and the risk of tumor recurrence or deterioration (11). A completely resected cystadenoma of the bile duct has a good prognosis and rarely recurs (12). In addition, the prognosis of patients with cystadenoma with a mesenchymal matrix is better than that of patients without a mesenchymal matrix (12).

In fact, as early as 2010 in the WHO classification of digestive system tumors (5th edition), BCA is no longer a disease type, but is divided into intraductal papillary neoplasms of the bile duct (IPNB) and mucinous cystic tumor (MCN) (13). But in clinical diagnosis, the concept of BCA is still used (14). We re-tested the estrogen receptors (ER) and progesterone receptors (PR) of the patient's specimen. The results showed that ER (-) and PR (-), suggesting that there is no ovarian-like stroma, so the patient cannot be diagnosed as MCN (13). BCA with negative ovarian stroma is likely to be IPNB (13).

IPNB is a rare tumor that was classified as a unique pathological entity in the WHO Classification of Digestive System Tumors (5th Edition) (13). IPNB can occur anywhere in the biliary tree, extrahepatic bile ducts (58%), intrahepatic bile ducts (33%), intrahepatic bile ducts (9%) are rare, and can be multifocal (15). In intrahepatic IPNB, affected bile duct dilatation is more common as cystic or aneurysm, while extrahepatic IPNB is cylindrical or fusiform dilatation (13). IPNB commonly shows a lack of ovarian-type stroma, prominent papillary proliferation with fibrovascular cores, and communication with prominent, cystically dilated bile duct (13). Most of the cystic structures in this case were not clearly lined with epithelium, but combined with symptoms and immunohistochemistry results of ER (-) and PR (-), we believe that this case is highly likely to be a cystic variant of IPNB. The treatment of IPNB is the same as that of BCA. The main choice for IPNB patients is radical surgery. The scope of surgery depends on the location of the tumor (16), rather than the unknown histopathological results before surgery (17).

The patient's liver function before the surgery was normal, but HBsAg(+), HBeAg(+), HBcAb(–), and HBV DNA levels (>20,000 IU/mL) were in line with the immune tolerance period of chronic hepatitis B (18). Chronic hepatitis B is known to increase the risk of liver failure, liver cancer, or cirrhosis, which may affect the prognosis of patients with biliary cystadenoma; however, more research is needed. Colorectal polyps are the most common precancerous lesions of colorectal cancer (19), and the removal of adenomatous polyps through colonoscopy can prevent colorectal cancer (20). After colonic polyps are removed, patients may still develop colon cancer eventually. Therefore, for this patient, postoperative health checks are very important.

## Data Availability Statement

The original contributions presented in the study are included in the article/supplementary material, further inquiries can be directed to the corresponding authors.

## Author Contributions

GH and TL provided cases and constructed a framework for reporting. YT sorted out the context of the case, summarized the characteristics of the case, and conducted a literature review. CW organized the images and participated in the literature review. SF provided new laboratory test results for this case. All authors contributed to the article and approved the submitted version.

## Conflict of Interest

The authors declare that the research was conducted in the absence of any commercial or financial relationships that could be construed as a potential conflict of interest.

## Publisher's Note

All claims expressed in this article are solely those of the authors and do not necessarily represent those of their affiliated organizations, or those of the publisher, the editors and the reviewers. Any product that may be evaluated in this article, or claim that may be made by its manufacturer, is not guaranteed or endorsed by the publisher.
